# Sleeping habits affect access to host by Chagas disease vector *Triatoma dimidiata*

**DOI:** 10.1186/s13071-016-1852-3

**Published:** 2016-11-03

**Authors:** Etienne Waleckx, Rafael Pasos-Alquicira, María Jesús Ramírez-Sierra, Eric Dumonteil

**Affiliations:** 1 Laboratorio de Parasitología, Centro de Investigaciones Regionales “Dr Hideyo Noguchi”, Universidad Autónoma de Yucatán, Mérida, México; 2 Department of Tropical Medicine, Tulane University, School of Public Health and Tropical Medicine, New Orleans, LA USA

**Keywords:** Triatomines, Chagas disease, Intrusion, Feeding, Domiciliation, Yucatán, Mexico

## Abstract

**Background:**

Chagas disease, caused by the parasite *Trypanosoma cruzi*, is mainly transmitted by blood-sucking bugs called triatomines. In the Yucatán Peninsula, Mexico, the main vector of *T. cruzi* is *Triatoma dimidiata*. While this species may colonize houses in other regions, it is mostly intrusive in Yucatán: it generally lives in sylvan and peridomestic areas, and frequently enters inside homes, likely attracted by potential vertebrate hosts, without establishing colonies. Bugs collected inside homes have a low nutritional status, suggesting that they cannot efficiently feed inside these houses. We hypothesized that this low nutritional status and limited colonization may be associated, at least in part, with the local practice in Mayan communities to sleep in hammocks instead of beds, as this sleeping habit could be an obstacle for triatomines to easily reach human hosts, particularly for nymphal instars which are unable to fly.

**Methods:**

We used an experimental chamber in which we placed a miniature bed in one side and a miniature hammock on the other side. After placing a mouse enclosed in a small cage on the bed and another one in the hammock as baits, *T. dimidiata* bugs were released in the chamber and their activity was video recorded during the night.

**Results:**

*T. dimidiata* adults and nymphs were able to reach the mouse in bed significantly more often than the mouse in hammock (Binomial test, *P* < 0.0001). Moreover, females reached the mice twice as often as did males. Most of the adult bugs reached the mouse in bed by walking, while they reached the mouse in hammock by flying. Nymphs presented a host-seeking index ten times lower than adult bugs and were also able, on a few occasions (4/132 released bugs), to reach the mouse in hammock.

**Conclusions:**

We conclude that sleeping in hammocks, as done in rural Yucatán, makes human hosts less accessible to the bugs. This, combined with other factors (e.g. absence of domestic animals sleeping inside houses), may explain, at least in part, the low nutritional status of bugs collected inside homes and the limited colonization of houses by *T. dimidiata* in the region. Nevertheless, while this sleeping habit limits contact with the bugs, it does not confer complete protection as adult bugs as well as some nymphs were still able to reach the host in hammock in our study.

## Background

Chagas disease, caused by the parasite *Trypanosoma cruzi*, is mainly transmitted to humans and other mammals by blood-sucking insects called triatomines, also known as kissing bugs. This disease is a major public health problem in the Americas, where six to seven million people are estimated to be infected with the parasite [1]. Current estimates suggest that there is a disease burden reaching 29,385,250 disease-adjusted life-years (DALYs), and health care costs of over $24,000 million are attributed to Chagas disease, which exceeds that of other global diseases such as rotavirus infection ($2000 million) or the cervico-uterine cancer ($4700 million) [2, 3]. In Mexico, 31 triatomine species are currently reported, and *T. cruzi* transmission is reported in most of the territory [4, 5]. One to two million people are generally estimated to be infected with *T. cruzi* in Mexico, but the more pessimist figures estimate that it could reach 5.5 million people [6, 7]. Despite this, Mexico has only a passive national surveillance program and no real national strategy to prevent vector-borne transmission of *T. cruzi* exists.

*Triatoma dimidiata* is one of the principal insect vectors of *T. cruzi*. This species as a large geographical distribution, ranging from Ecuador to Southern Mexico [8], and it is the main vector reported in the Yucatán Peninsula in Mexico. While *T. dimidiata* may colonize houses in other regions, particularly in Central America [9, 10], this species is mostly intrusive in Yucatán: it lives generally in sylvan and peridomestic areas, and frequently enters inside homes, likely attracted by artificial light and potential vertebrate hosts, without establishing colonies [11–13]. Moreover, bugs collected inside homes have a low nutritional status, suggesting that they cannot efficiently feed inside these houses [14].

The objective of the current study was to test the hypothesis that this low feeding status and limited colonization may be associated, at least in part, with the local practice in Mayan communities to sleep in hammocks instead of beds, as this sleeping habit could be an obstacle for triatomines to easily reach human hosts, particularly for nymphal instars which are unable to fly, thus affecting the accomplishment of their life-cycle.

## Methods

### *Triatoma dimidiata* specimens

Males, females, and N5 nymphs (fifth-instar nymphs) of uninfected laboratory-reared *T. dimidiata* originating from triatomines collected in the field within the framework of experimental vector control interventions [15, 16] in the rural villages of Bokobá (21°00’27”N, 89°10’47”W), Teya (21°02’55”N, 89°04’25”W) and Sudzal (20°52’19”N, 88°59’20”W), were used. The bugs, maintained by feeding every 2–3 weeks on pigeons, were starved for 10–15 days before being used in the experiments.

### *Triatoma dimidiata* host-seeking behaviour assay

**Fig. 1:**
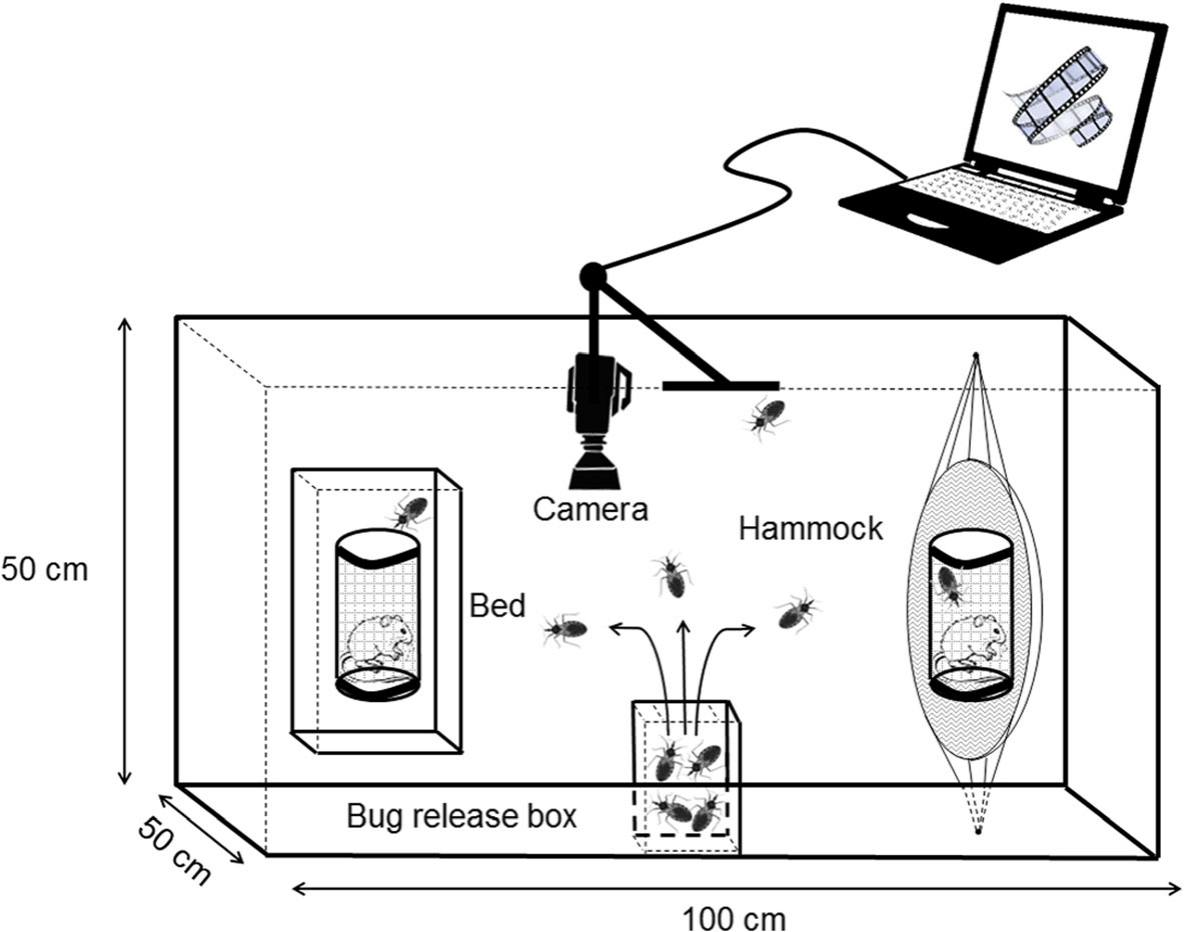
Experimental system. Upper view

To observe the host-seeking behaviour of *T. dimidiata*, we used an experimental chamber consisting of a rectangular arena (0.5 × 1 × 0.5 m) made with a wooden floor, plastic insect screen walls, and a glass ceiling, in which we placed a miniature bed in one side and a miniature hammock on the other side (Fig. 1). After placing a mouse enclosed in a cage on the bed and another one in the hammock, we placed a small cardboard box containing *T. dimidiata* bugs, and serving as a refuge, in the middle of the chamber at dusk. Bugs could then exit the cardboard refuge and forage freely within the chamber until the next morning. The activity of the bugs was video recorded (7 pm–7 am) to measure the number and time of accesses to both mice, and how each access occurred (i.e. flying or walking) (Fig. 1). This assay was repeated for 18 nights (five nights with ten males, five nights with ten females, four nights with eight N5 nymphs, and four nights with 25 N5 nymphs). The positions of the bed and the hammock were switched between each experiment to avoid any bias.

### Data analysis

The host-seeking index was defined as the ratio between the number of accesses to both mice and the total number of bugs released. The proportions of bugs which reached the mouse in bed *vs* hammock were compared by using binomial tests and the association between the sleeping location of the mice (bed *vs* hammock) and how these were reached by adult bugs (walking *vs* flying) was tested using the Chi-square (*χ* ^2^) test. In all cases, *P* < 0.05 was considered statistically significant.

## Results

### Activity of *Triatoma dimidiata* bugs

**Fig. 2:**
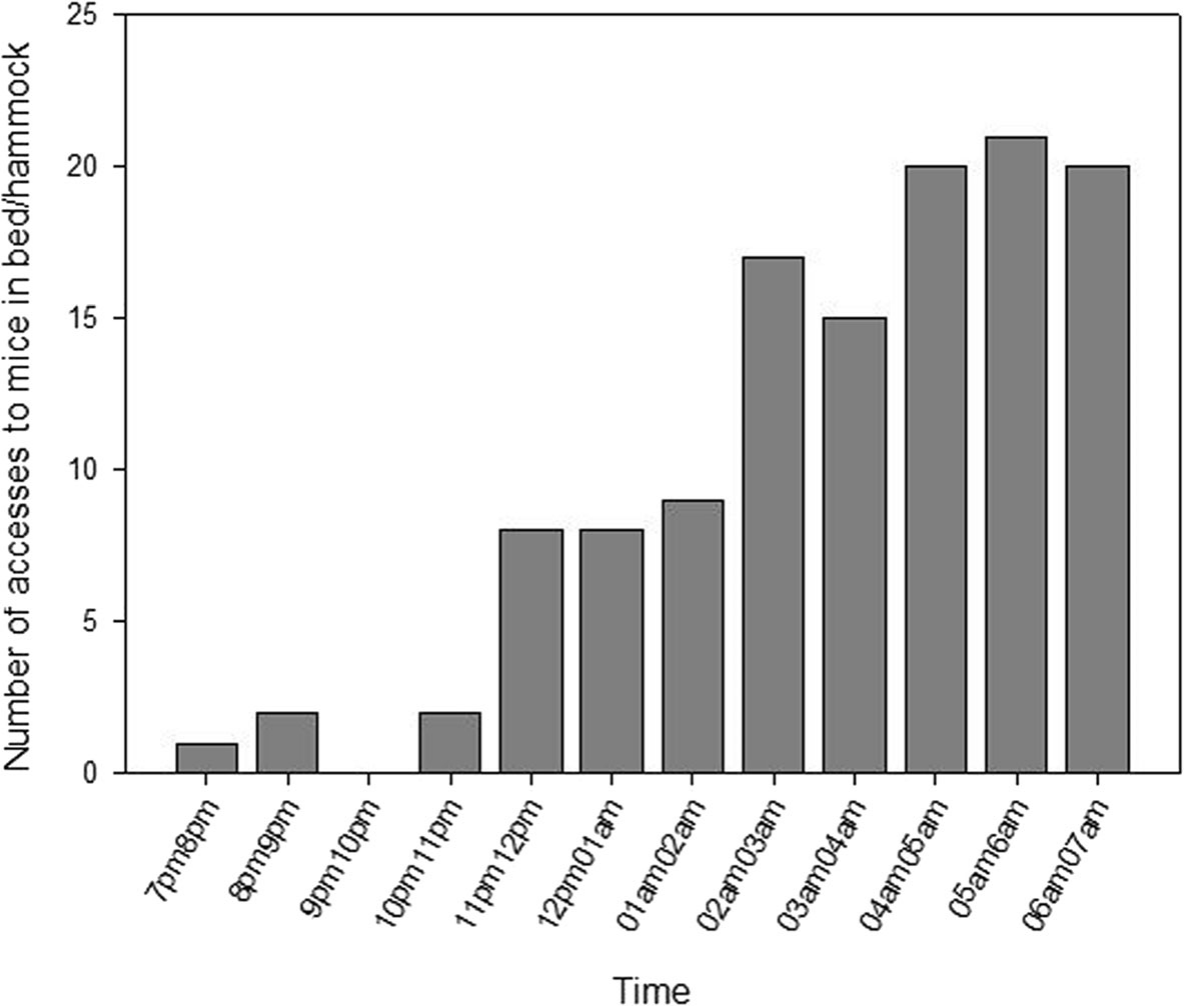
Number of accesses to mice in bed/hammock as a function of time

In total, 232 bugs, including 50 males, 50 females, and 132 N5 nymphs were released over the 18 nights of the experiment. Mice were reached 123 times, giving a global host-seeking index of 0.53 (123/232, Table 1). Females reached the mice twice as often as did males (host-seeking index, 1.44 and 0.68, respectively). Moreover, the N5 nymph host-seeking index (0.13) was almost 10 times lower than the adult index (1.06, Table 1). Finally, we observed an increase of the number of accesses to both mice as the night progressed (Fig. 2). Accesses to mice were observed for all but one (9 to 10 pm) 1-h time intervals among the 18 nights of the experiment, ranging from 1 access between 7 and 8 pm to 21 accesses between 6 and 7 am.

**Table 1:** Host-seeking index of the bugs by sex and stage

	Total no. of bugs released	No. of times bugs accessed to mice in bed/hammock	Host-seeking index^a^
Males	50	34	0.68
Females	50	72	1.44
N5 nymphs	132	17	0.13
Total	232	123	0.53

^a^The host-seeking index was defined as the ratio between the number of times bugs accessed to mice in bed/hammock and the total number of bugs released

*Abbreviation*: *N5* fifth-instar

### Bed *vs* hammock

**Fig. 3:**
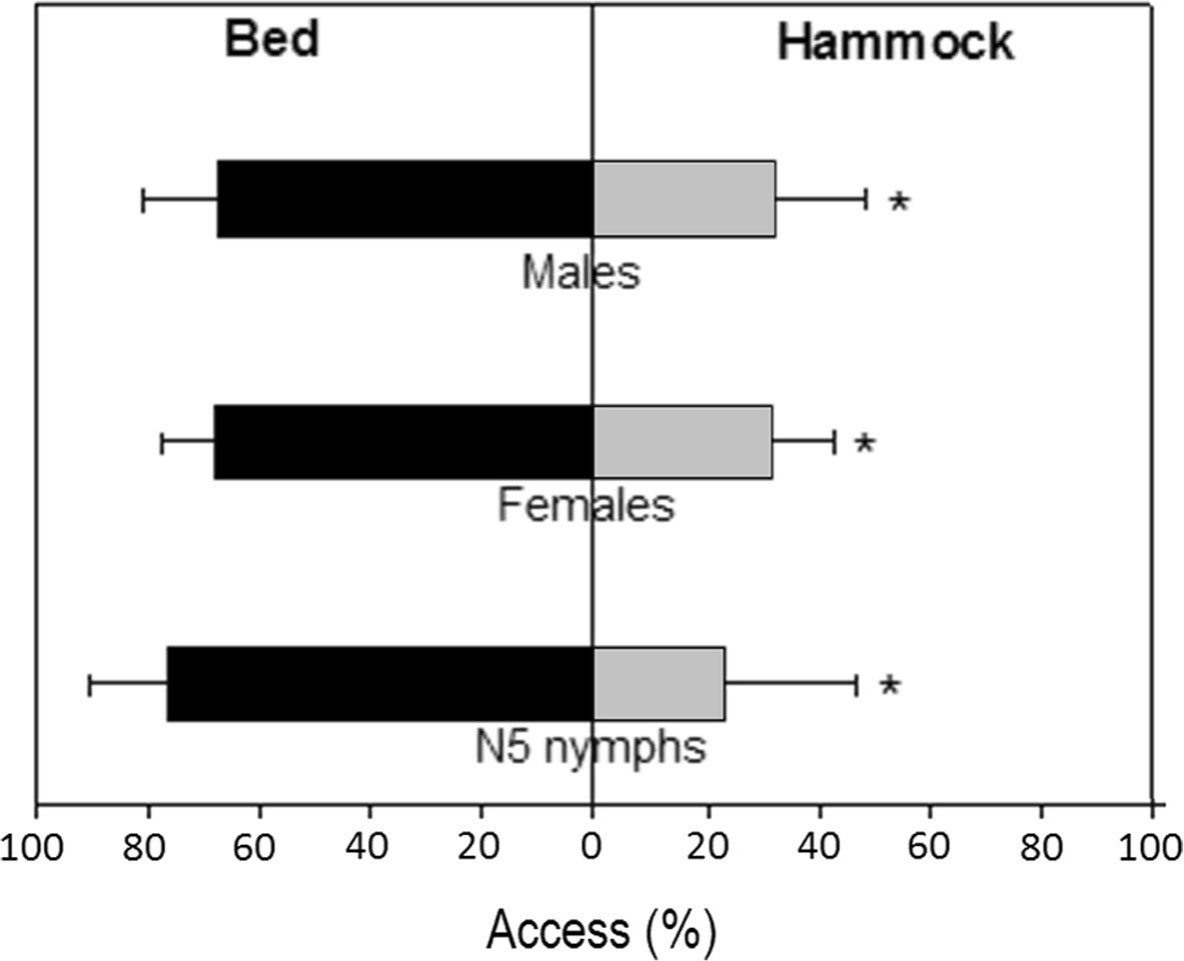
Percentage of access to mouse in bed *vs* hammock. Error bars represent the upper limit of the 95 % confidence interval, according to Newcombe [17], without correction for continuity. *indicates a significant difference between bed and hammock (Binomial tests; males: *P* = 0.029; females: *P* = 0.0015; N5 nymphs: *P* = 0.0245)

As observed in Fig. 3, released bugs reached the mouse in bed two to three times more frequently than the mouse in hammock. Of a total of 123 recorded accesses to hosts, 85 (69.1 %) were done to the mouse in bed. The difference between the access to mice in bed *vs* hammock was significant for all bugs taken together, as well as for each stage and sex separately (Binomial tests, *P* < 0.05 in all cases). The effect tended to be more pronounced for N5 nymphs (access to bed: 76.5 % *vs* access to hammock: 23.5 %) than for adult bugs (67.9 *vs* 32.1 %), but this tendency did not reach significance (*χ* ^2^ = 0.5012, *df* = 1, *P* = 0.479). Interestingly, nymphs were also able, on a few occasions (4/132 released bugs), to reach the mouse in hammock by walking along the arms of the hammock.

### Walking *vs* flying

**Fig. 4:**
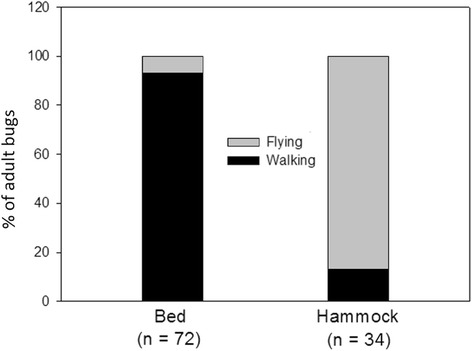
Proportion of adult bugs which walked/flew to the bed/hammock (*n*, total number of adult bugs which reached the mice in bed/hammock)

More than 93 % (67/72) of the recorded accesses of adult bugs to the mouse in bed were by walking, while the mouse in hammock was reached by flying adults in 88 % (30/34) of cases (Fig. 4). There was a significant association between the sleeping location of the mice (bed *vs* hammock) and how they were reached by adult bugs (walking *vs* flying) (*χ* ^2^ = 69.04, *df* = 1, *P* < 0.0001).

## Discussion

The level of domiciliation/intrusion of triatomine species is a major determinant of their vectorial capacity and has important implications for the design of effective vector control interventions [18]. In the Yucatán Peninsula, Mexico, *T. dimidiata* is responsible for a seroprevalence of *T. cruzi* infection in humans of up to 4–5 % in rural areas [19]. In this region, the intrusive behaviour of *T. dimidiata*, characterized by an infestation of human dwellings by sylvatic and peridomestic adult bugs on a seasonal basis without establishment of colonies, is well documented [11, 12, 20–23]. This seasonal intrusion into human dwellings makes control efforts challenging in Yucatán. For instance, modelling studies have suggested that vector control interventions based on insecticide spraying would not be suitable, particularly because of the necessity to spray insecticide yearly during a reduced period of time in the year, almost operationally impossible [24]. Alternative control intervention strategies, based on the Ecohealth principles, have recently been implemented at different pilot scales [15, 16, 25] and are currently giving promising results (Waleckx et al., unpublished data). While *T. dimidiata* generally does not establish colonies inside human dwellings in the Yucatán Peninsula, in other parts of its geographical distribution, such as in Guatemala, populations are well domiciliated, as evidenced by bug collections throughout the country showing high infestation and colonization indexes [10, 26].

The limited colonization of human dwellings in the Yucatán Peninsula has been proposed to be related to a low fecundity and high mortality resulting from poor feeding of bugs inside houses [14]. This may be explained by the fact that domestic animals are generally kept close to but outside dwellings in the region [12]. Nevertheless, other factors may also be involved and here, we particularly investigated the role of the local practice to sleep in hammocks instead of beds.

In our study, we first observed that *T. dimidiata* females gained access to mice in bed/hammock twice more than did males. This observation may be explained by a higher voracity of females, as reported by Zeledon et al. [27]. In their study, Zeledon et al. did not compare the voracity of nymphal instars with that of adults, but our results suggest that N5 nymphs are less voracious as they acceded to the mice 10 times less than adult bugs.

Foraging of triatomine bugs for hosts is believed to occur mostly early in the night, while the search for refuges occurs later at night, supposedly after feeding [28]. Here, we observed that host-seeking increased as the night progressed. A similar pattern has already been reported for *T. dimidiata* under experimental conditions [13, 29]. It is unclear if this is specific to this species, or if it is due to the different experimental systems, in which the bugs were not able to feed (e.g. in the current experiment, mice were placed inside small cages protecting them from the bites). Bugs may thus keep foraging actively for a much longer period of time in an attempt to reach the detected host. Nevertheless, bugs may also have difficulty in finding available hosts in natural conditions and may present an activity pattern similar to the one observed here. Alternative experimental systems may be developed in future studies to further explore the host-seeking behaviour of *T. dimidiata*.

Importantly, we found in our study that bugs were able to gain significantly more access to the mouse in bed compared to the mouse in hammock, suggesting that the latter makes the host less accessible to the bugs. This “hammock effect” tended to be higher for N5 nymphs, which are unable to fly, and a significant association between the location of the mice and the way they were reached (almost 80 % of accesses to the host in hammock were done by flying bugs) was found. The observed hammock effect might even be more pronounced for younger nymphal instars, which are believed to have a reduced activity compared to older nymphal instars and adult bugs. If confirmed, the latter hypothesis is interesting because it means that even if a female releases eggs in a human dwelling in an attempt to colonize, young nymphs might have difficulty feeding, leading to their death, and thus preventing successful colonization of human dwellings. In this sense, a very low survival of nymphal instars of *T. dimidiata* was previously predicted by mathematical models fitted to *T. dimidiata* demographic data in the Yucatán Peninsula [21].

## Conclusions

The results of our study suggest that sleeping in hammocks, as is common practice in rural Yucatán, makes human hosts less accessible to triatomine bugs. This, combined with other factors (e.g. absence of domestic animals sleeping inside houses), may explain at least in part, the low nutritional status of bugs collected inside houses and the subsequent limited colonization of homes by *T. dimidiata* in the region. Nevertheless, while this sleeping habit limits contacts with the bugs, it does not confer complete protection as adult bugs as well as N5 nymphs were still able to reach the host sleeping in hammock in our study. Confirmation of these findings in the field would be interesting but may be difficult to perform because of ethical issues.

In the Yucatán Peninsula, Mexico, sleeping in hammock is an important cultural practice, primarily because hammocks are more comfortable than beds in this very warm region as they allow a best air flush. While Mayan communities do not associate this practice with vector protection [12, 30], it could be promoted as part of Ecohealth vector control strategies. Indeed, it could help prevent the colonization of human dwellings by *T. dimidiata* as well as help limit the exposition of human populations to triatomine bites and Chagas disease transmission.

## Abbreviations

DALYs: Disease-adjusted life-years
N5: Fifth-instar nymph
